# Persistent Gut Microbiota Dysbiosis in Pediatric Crohn’s Disease: A Next-Generation Sequencing Pilot Study

**DOI:** 10.3390/biom16060801

**Published:** 2026-05-29

**Authors:** Georgia Valentina Tita, Cristina Rebeca Fogas, Kinga Cristina Slavescu, Vasile Marcel Tantau, Stefana Arlinda Medan, Daniela Elena Serban

**Affiliations:** 1 3rd Medical Discipline, Department of Internal Medicine, “Iuliu Hatieganu” University of Medicine and Pharmacy, 400012 Cluj-Napoca, Romania; georgia.tartamus@umfcluj.ro (G.V.T.); fogas.cristina.rebeca@elearn.umfcluj.ro (C.R.F.); 2 Department of Mother and Child, 2nd Clinic of Pediatrics, Emergency Clinical Hospital for Children, “Iuliu Hatieganu” University of Medicine and Pharmacy, 400177 Cluj-Napoca, Romania; kinga.slavescu@umfcluj.ro; 3 Department of Internal Medicine and Gastroenterology, “Prof. Dr. Octavian Fodor” Regional Institute of Gastroenterology and Hepatology, “Iuliu Hatieganu” University of Medicine and Pharmacy, 400162 Cluj-Napoca, Romania; marcel.tantau@umfcluj.ro; 4 Department of Anatomy and Embryology, “Iuliu Hatieganu” University of Medicine and Pharmacy, 400006 Cluj-Napoca, Romania; tartamus.stefana.arlinda@elearn.umfcluj.ro

**Keywords:** Crohn’s disease, gut microbiome, diversity, metagenomics

## Abstract

**Background:** Crohn’s disease (CD) is characterized by gut microbiota alterations including reduced microbial diversity, loss of commensal species, and increased abundance of opportunistic taxa. **Methods:** This prospective study was conducted between 2022 and 2024 at the Emergency Clinical Hospital for Children, Cluj-Napoca. Children with CD and healthy controls were evaluated. The gut microbiota was analyzed using shotgun metagenomics. Bioinformatic processing assessed alpha and beta diversity, core microbiome composition, and differential taxa. **Results:** Ten patients with CD and eight healthy children were included; five patients were re-evaluated after a median interval of 14 weeks. The Shannon index was significantly lower in CD patients compared with controls (*p* = 0.037). Beta diversity analysis suggested partial separation between CD at diagnosis and controls (*p* = 0.041). An inverse correlation was observed between the Shannon index and the clinical score (*p* = 0.028). *Ruminococcus gnavus* was among the taxa contributing to group separation. At follow-up, all patients were in clinical remission, while 80% had achieved biological remission and mucosal healing. They showed persistently reduced alpha diversity and distinct microbial communities compared with controls (*p* = 0.028 and *p* = 0.005, respectively). **Conclusions:** Pediatric CD was correlated with dysbiosis that persisted despite remission. Reduced alpha diversity was associated with greater disease severity at diagnosis.

## 1. Introduction

Crohn’s disease (CD) is a form of inflammatory bowel disease (IBD) characterized by chronic transmural inflammation that may involve any segment of the gastrointestinal tract, with a relapsing–remitting course [1].

Pediatric CD differs from adult-onset disease in several important aspects, being more commonly characterized by extensive disease involvement, a more rapid early progression, and a higher risk for the development of complicated phenotypes over time [2]. In contrast to adults, where disease burden is primarily related to intestinal damage, pediatric CD has additional systemic consequences due to the impact of chronic inflammation on growth and development. Malnutrition, growth failure, and delayed puberty are reported in children and may significantly influence long-term outcomes [3]. Furthermore, while quality of life is affected in both age groups, pediatric patients experience unique challenges related to school performance, social functioning, and emotional development [4].

IBD results from a complex interplay between genetic susceptibility, epigenetic modifications, environmental factors, intestinal barrier dysfunction, and an aberrant immune response [5].

Significant alterations in the gut microbiota composition have been described in patients with CD characterized by reduced microbial diversity and abundance, loss of commensal species with anti-inflammatory properties (such as *Faecalibacterium prausnitzii*), and increased abundance of opportunistic taxa (such as *Escherichia coli*) [6]. Regarding the evolution of the gut microbiota in IBD patients undergoing treatment, current evidence suggests that none of the available therapeutic interventions fully restore a state of eubiosis [7]. For CD, the use of exclusive enteral nutrition (EEN) may lead to a reduction in microbial diversity and depletion of key commensal species during therapy, alongside a decrease in pro-inflammatory taxa [8]. Similarly, anti-TNF therapies are associated with partial normalization of microbial composition; however, complete restoration to a profile comparable to that of healthy individuals remains unlikely [9].

In recent years, treatment strategies in CD have shifted toward a treat-to-target approach, aiming not only for clinical remission (CR), but also for biological remission (BR), mucosal healing (MH), and potential transmural healing (TH) [10,11]; however, current treat-to-target frameworks do not incorporate gut microbiota assessment, despite evidence supporting its role in disease pathogenesis, prognosis, and therapeutic response.

Traditional methods for microbiota analysis, such as 16S rRNA sequencing and qPCR, provide limited characterization, typically at the genus level, and do not accurately capture fine taxonomic and functional diversity. In contrast, next-generation shotgun metagenomic sequencing enables detailed species-level identification and in-depth analysis of microbial functional potential, significantly enhancing the understanding of dysbiosis in IBD [12].

Despite growing interest in the gut microbiota as a potential biomarker, longitudinal data in pediatric CD, particularly using metagenomic approaches, remain scarce. This gap limits the current understanding of microbiota dynamics during treatment and represents a major barrier to incorporating microbiota-based parameters into treat-to-target strategies. In addition, data from pediatric IBD patients in Romania are lacking. Therefore, this study aims to characterize the intestinal microbiota in children with CD from Romania and to explore its evolution following remission induction therapy.

## 2. Materials and Methods

### 2.1. Study Population

A prospective analytical study was conducted between January 2022 and June 2024 at the Emergency Clinical Hospital for Children Cluj-Napoca (ECHC Cluj-Napoca). The study cohort comprised two groups.

The first group included pediatric patients (under 18 years old) diagnosed at ECHC Cluj-Napoca with CD according to the ESPGHAN Porto criteria [13]. Eligible patients were newly diagnosed and presented with active disease, defined clinically by wPCDAI ≥ 12.5, biologically by C reactive protein [CRP] ≥ 0.6 mg/dL and/or erythrocyte sedimentation rate [ESR] ≥ 20 mm/h, microscopically by fecal calprotectin [FC] ≥ 250 μg/g and endoscopically by active mucosal lesions at ileocolonoscopy and/or upper endoscopy. Exclusion criteria included acute intestinal infections, treatment before diagnosis, and inability to obtain a fecal sample prior to bowel preparation for diagnostic colonoscopy.

The second group consisted of healthy age- and sex-matched controls (<18 years old), recruited prospectively as a comparator group for gut microbiota analyses. None had clinical manifestations suggestive of IBD, a history of chronic gastrointestinal disorders, or other major chronic diseases.

Patients with CD were evaluated at study inclusion and at a subsequent follow-up visit (the first intestinal imaging reassessment performed in the absence of clinical disease activity). Treatment type and duration were determined individually by the treating physician, in accordance with current guidelines [1,14,15,16]. Nutritional counseling was provided by a specialized dietitian for patients undergoing the Crohn’s Disease Exclusion Diet (CDED).

### 2.2. Diagnostic and Follow-Up Assessment

Data were systematically collected and entered into a prospectively maintained study database. Variables included age, sex, area of residence, disease phenotype, treatment details (induction and maintenance therapy), levels of serum inflammatory markers (CRP, ESR) and fecal markers (FC), disease activity score (weighted Pediatric Crohn’s Disease Activity Index [wPCDAI]), and intestinal ultrasound findings.

Disease phenotype was classified according to the Paris classification [17]. Clinical disease activity was assessed using the wPCDAI and categorized as mild (12.5–40), moderate (>40–57.5), or severe (>57.5). CR was defined as wPCDAI < 12.5 [18]. Biological activity was indicated by CRP ≥ 0.6 mg/dL and/or ESR ≥ 20 mm/h, whereas biological remission (BR) corresponded to values below these thresholds [19].

FC levels were measured using a chemiluminescence assay at the ECHC Cluj-Napoca laboratory or Bioclinica Laboratories (Cluj-Napoca, Romania). FC values ≥ 250 μg/g were considered indicative of microscopic activity, while MH was defined as FC values below 250 μg/g [20].

Intestinal ultrasound, frequently performed as hydrosonography, was used to assess intestinal and peri-intestinal changes, as well as imaging activity in CD. Examinations were conducted in the Radiology and Imaging Department of ECHC Cluj-Napoca by experienced radiologists specialized in gastrointestinal imaging. Imaging activity was defined by the following criteria: bowel wall thickness (BWT) ≥ 3 mm, blurred stratification with submucosal thickening or complete loss of stratification, presence of color/power Doppler signal, intraperitoneal collections, mesenteric fat proliferation, and regional lymphadenopathy [21]. Imaging remission (IR) was defined as BWT < 3 mm and absence of Doppler signal [22]. TH was defined as the simultaneous presence of MH and IR.

### 2.3. Gut Microbiota Sampling and Bioinformatic Analysis

For gut microbiota analysis, fecal samples were collected either in the hospital setting or at home, in a volume of approximately 0.5–1 cm^3^, or approximately 1 g, prior to bowel cleansing (enema or laxative preparation) for endoscopic procedures and before initiation of remission induction therapy. Immediately after collection, samples were transferred to a nucleic acid stabilization medium, namely DNA/RNA Shield™ Collection Tube (Zymo Research, Irvine, CA, USA) and maintained according to the manufacturer’s recommendations, allowing ambient-temperature storage and transport without special handling requirements.

Gut microbiota analysis was performed at SYNLAB laboratories (Esplugues de Llobregat, Spain) using the commercial myBIOME platform based on next-generation shotgun metagenomic sequencing (NGS) [23]. The laboratory report provided to the authors did not include detailed sequencing metrics such as sequencing depth, read quality statistics, filtering thresholds, or reference database versions. In one case, metagenomic analysis could not be performed due to an excessive proportion of human DNA in the collected sample.

Age and sex were recorded for the control group, and gut microbiota analysis was performed according to the same methodology.

For microbiome data analysis, relative abundance tables (%) at the phylum and taxon levels, generated from the microbiome sequencing output, were used directly. Low-abundance and low-variance features were filtered using standard parameters. Singleton features were removed to reduce spurious variability. Data were normalized using total sum scaling (TSS) to account for differences in sequencing depth. Relative abundance was assessed both at the phylum level and for dominant taxa. Phyla with very low relative abundance (Desulfobacterota A, Verrucomicrobiota, Synergistota, Cyanobacteria, Euryarchaeota, Fusobacteriota, Ascomycota, and unclassified Eukaryota) were excluded from comparative analyses due to their low frequency and limited biological relevance.

Alpha diversity was evaluated using the Shannon diversity index and beta diversity was analyzed using principal coordinates analysis (PCoA) based on the Bray–Curtis dissimilarity index, with statistical significance assessed using PERMANOVA and pairwise PERMANOVA tests. Core microbiome analysis was performed at the taxon level using a minimum relative abundance threshold of 0.1% and a minimum prevalence of 50% within each group. A heatmap of relative abundance was generated to visualize sample clustering and the distribution of major taxa across groups.

Differential taxa were identified using LEfSe (Linear Discriminant Analysis Effect Size), including calculation of *p*-values, LDA (Linear Discriminant Analysis) scores, and false discovery rate (FDR) correction for multiple testing. FDR correction was performed using the Benjamini–Hochberg procedure to reduce the likelihood of false-positive findings resulting from multiple simultaneous comparisons across microbial taxa. Random Forest classification was used as an exploratory analysis to rank taxa according to their contribution to group discrimination, using 500 trees and standard randomization parameters; model performance was evaluated using the out-of-bag (OOB) error estimate. The results are presented as comparative plots and descriptive statistics.

Given the small sample size, subgroup analyses based on treatment were not performed.

### 2.4. Statistical Analysis

The distribution of continuous variables was assessed using the Shapiro–Wilk test, and data are reported as median and interquartile range (IQR). Categorical variables are expressed as absolute frequencies and percentages. Comparisons of continuous variables between groups were performed using the Mann–Whitney U test or the Kruskal–Wallis test, while within-subject comparisons were conducted using the paired Wilcoxon test. Correlations were assessed using Spearman’s rank correlation coefficient at the time of diagnosis. A *p*-value < 0.05 was considered statistically significant.

Statistical analyses were performed using Microsoft Excel (Microsoft Corporation, Redmond, WA, USA), R software (version 4.4.2; R Core Team, Vienna, Austria) with the RStudio interface (version 2025.05; Posit Software, Boston, MA, USA), and the online platform MicrobiomeAnalyst (Xia Lab, McGill University, Montreal, QC, Canada).

### 2.5. Ethical Considerations

The study protocol was conducted in accordance with the principles of the Declaration of Helsinki. Medical data were used only after written informed consent had been acquired from each participant and their legal guardian(s). Preliminary approval was obtained from the Clinical Studies Quality Assurance Committee of ECHC Cluj-Napoca (No. 81 SC/20 May 2021), as well as from the Ethics Committee of the “Iuliu Hațieganu” University of Medicine and Pharmacy, Cluj-Napoca (No. AVZ99/3 May 2022).

## 3. Results

### 3.1. Study Population Characteristics

A total of 10 pediatric patients with CD and 8 healthy children, serving as controls, were included in the study. Follow-up fecal samples were successfully collected from 5 patients with CD.

At diagnosis, the median age of patients with CD was 14.5 years old (IQR: 12.9–16.0), ranging from 6.1 to 17.7 years old. Sex distribution showed a male predominance (80%). Regarding the area of residence, the distribution was relatively balanced, with 40% of patients from urban areas and 60% from rural areas.

Phenotypic disease characteristics, including age at diagnosis, disease location, behavior, and growth status, are summarized in Table 1.

Assessment of clinical disease activity at diagnosis using the wPCDAI showed that one patient (10%) had no clinical manifestations, while 40% had mild disease, 20% had moderate disease, and 30% had severe disease.

At diagnosis, median values were as follows: wPCDAI 23.8 (IQR: 18.1–51.3), ESR 36 mm/h (IQR: 22.5–55.0), CRP 5.0 mg/dL (IQR: 2.1–6.5), FC 1094 μg/g (IQR: 989–1631), and BWT 4.35 mm (IQR: 4.0–6.7).

Regarding remission induction therapy, corticosteroid monotherapy was used in 1 patient (10%), combined corticosteroid and nutritional therapy in 4 patients (40%), corticosteroids with nutritional therapy and azithromycin in 1 patient (10%), nutritional therapy alone in 2 patients (20%), and nutritional therapy plus 5-aminosalicylic acid (5-ASA) in 2 patients (20%). Nutritional management included CDED in all patients and was preceded by EEN in 60% of cases, with a median duration of 2.5 weeks. Adherence to CDED was intermittent in one case. None of the patients received biological therapy during the induction phase. For maintenance of remission, azathioprine monotherapy was the most frequently used regimen, prescribed in 60% of patients. Combination therapy with azathioprine plus 5-ASA was used in 20% of patients. 5-ASA monotherapy and methotrexate monotherapy were each administered in 10% of patients. Overall, azathioprine-containing regimens were used in 80% of cases.

The median time interval between diagnosis and follow-up was 14 weeks (IQR: 12–18), ranging from 9 to 44 weeks. In all re-evaluated patients, remission induction therapy included CDED, combined with EEN in 1/5 cases (20%), corticosteroids in 2/5 cases (40%), or 5-ASA in 2/5 cases (40%).

At follow-up, all patients were in CR (wPCDAI < 12.5), while 80% achieved BR and the same proportion achieved MH. IR was observed in 60% of patients, and among those with persistent lesions, 40% showed ultrasonographic improvement. TH was observed in 40% of cases.

The control group consisted of healthy children (*n* = 8), predominantly male (62.5%), with a median age of 13.4 years (IQR: 12.1–16.05).

### 3.2. Between-Group Microbial Analysis

The relative abundance of the main bacterial phyla is presented in Table 2. The abundance of Firmicutes and Bacteroidota differed significantly between healthy children and patients with CD (*p* = 0.02 and *p* = 0.015, respectively). Firmicutes were decreased and Bacteroidota increased in CD, both at diagnosis and follow-up, without significant changes over time (*p* = 0.43 and *p* = 0.67, respectively). Actinobacteriota and Proteobacteria did not differ significantly between groups.

To assess taxon-level differences in the microbiota, a differential abundance analysis was performed, comparing healthy children with patients with CD at diagnosis and at follow-up.

In the comparison between healthy children and patients with CD at diagnosis, several species within the phylum Firmicutes were significantly depleted in CD: *Agathobacter rectale* (median difference: −0.078; *p* = 0.004), *Fusicatenibacter saccharivorans* (median difference: −0.020; *p* = 0.020), and *Ruminococcus bromii* (median difference: −0.035; *p* = 0.035). In contrast, *Ruminococcus gnavus*, also belonging to the phylum Firmicutes, was significantly more abundant in patients with CD (median difference: 0.004; *p* = 0.028). Among Bacteroidota, *Bacteroides stercoris* was significantly reduced in CD at diagnosis (−0.037; *p* = 0.026).

**Figure 1:**
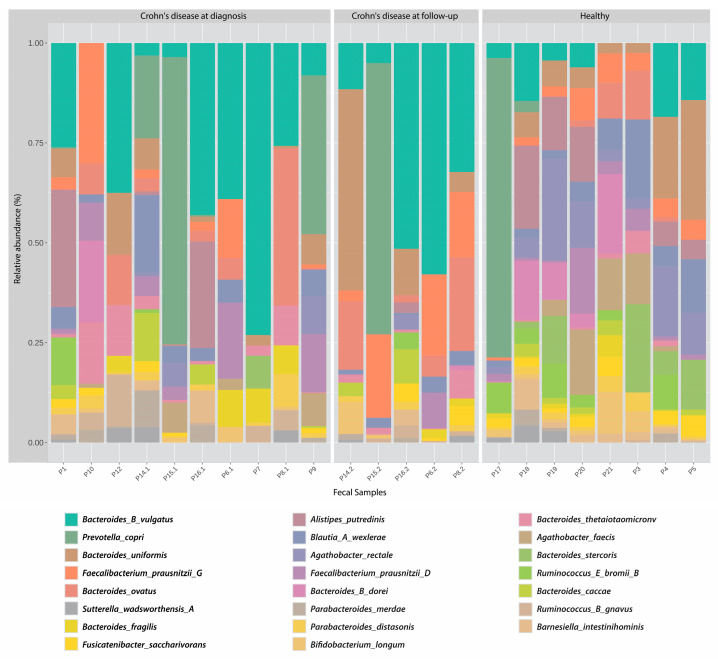
Relative abundance of the main bacterial taxa in patients with Crohn’s disease (CD) at diagnosis and at follow-up, and in healthy children.

In the comparison between healthy children and patients with CD at follow-up, *Alistipes putredinis* (Bacteroidota) was more abundant in healthy individuals, with borderline statistical significance (*p* = 0.054). Other core commensal species (*Bifidobacterium longum*, *Bifidobacterium caccae*, *Bifidobacterium fragilis*) were also less abundant in CD, although without statistical significance (*p* > 0.05). No significant species-level differences were identified between patients with CD at diagnosis and those at follow-up. The relative abundances of the main taxa in patients with CD at diagnosis, at follow-up, and in healthy controls are illustrated in Figure 1.

For total *Faecalibacterium prausnitzii*, defined as sum of all subtypes, the median relative abundance was 3.97% (IQR: 3.21–4.96) in healthy children, 5.28% (IQR: 0.97–6.63) in patients with CD at diagnosis, and 10.2% (IQR: 1.71–11.8) at follow-up. No statistically significant differences were observed between groups.

**Figure 2:**
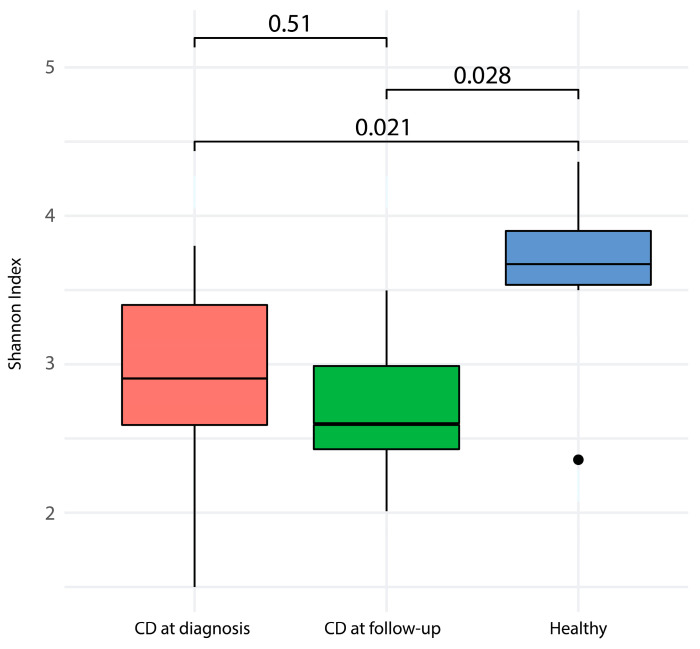
Comparison of alpha diversity (Shannon index) between patients with CD at diagnosis and at follow-up, and healthy children (including *p*-values).

The Shannon index (alpha diversity) was significantly lower in patients with CD, both at diagnosis and at follow-up, compared to healthy children (*p* = 0.021 and *p* = 0.028, respectively), with no significant changes between the two time points within the CD group (*p* = 0.51). A graphical representation of these differences is shown in Figure 2. In the healthy control group, the Shannon index had a median value of 3.68 (IQR: 3.53–3.89).

**Figure 3:**
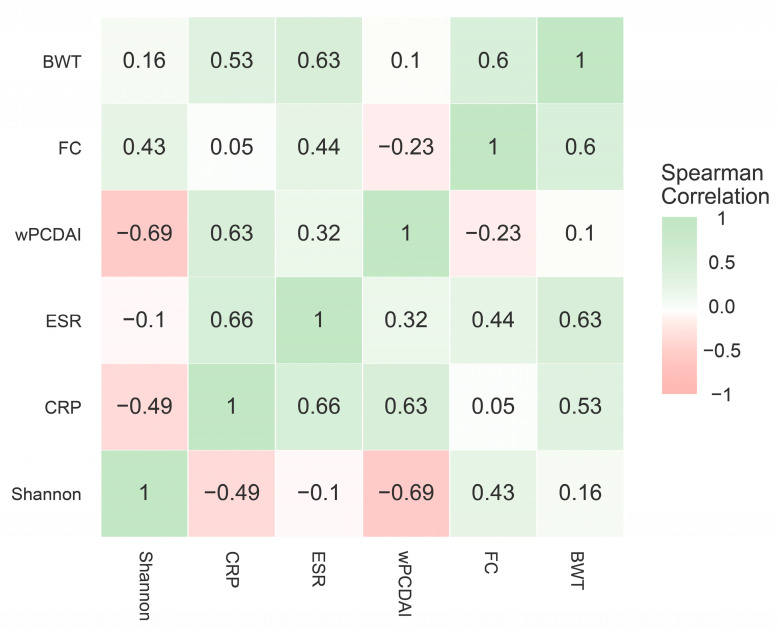
Spearman correlation matrix between the Shannon index and clinical scores and inflammatory markers in CD at diagnosis (BWT: bowel wall thickness, FC: fecal calprotectin, wPCDAI: weighted Pediatric Crohn’s Disease Activity Index, ESR: erythrocyte sedimentation rate, CRP: C reactive protein).

The association between the Shannon index and clinical and inflammatory parameters was evaluated using Spearman correlation analysis (Figure 3). A significant inverse correlation was observed between Shannon diversity index and the wPCDAI score (ρ = −0.687, *p* = 0.028), suggesting that lower microbial diversity was associated with more severe clinical disease activity. Correlations with other inflammatory markers, including CRP, ESR, FC, and BWT, were not statistically significant.

**Figure 4:**
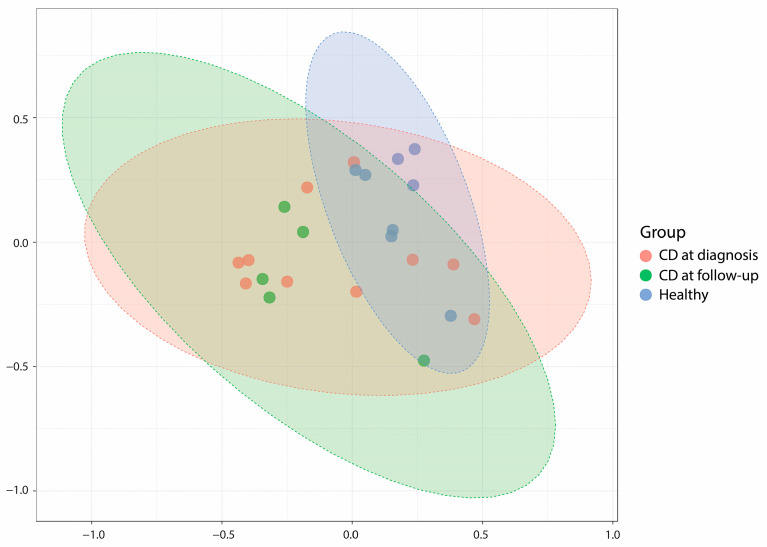
Principal coordinates analysis (PCoA) of gut microbiota beta diversity (each point represents an individual sample; shaded ellipses indicate the confidence regions for each group).

Beta diversity analysis, based on Bray–Curtis dissimilarity and PCoA, showed that healthy individuals and patients with CD at follow-up had significantly different gut microbial communities (PERMANOVA *p* = 0.005, FDR = 0.015, R^2^ = 19.7%). Partial separation was also observed between healthy individuals and patients with CD at diagnosis (*p* = 0.041, FDR = 0.0615). In contrast, no significant differences in beta diversity were detected between patients with CD at diagnosis and those at follow-up (*p* = 0.808, FDR = 0.808), indicating limited changes in overall microbial community structure over the follow-up period. The graphical representation of the PCoA analysis is shown in Figure 4.

**Figure 5:**
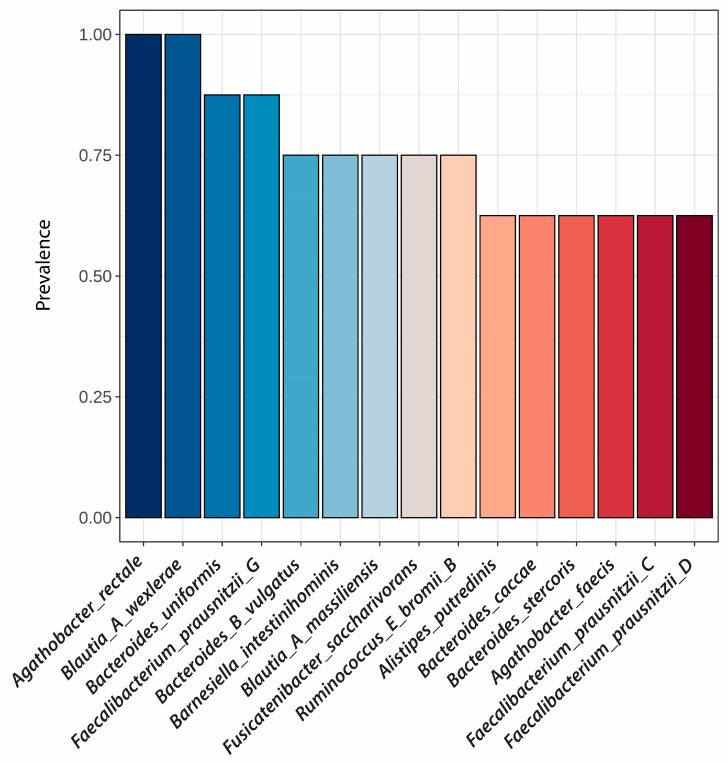
Core microbiome of the control group. Taxa are presented along with their prevalence (% of samples in which each taxon was detected).

Core microbiome analysis in the control group identified 15 taxa present in at least 50% of samples. The most prevalent members of the core microbiota included *Blautia wexlerae* and *Agathobacter rectale*, detected in 100% of samples, as well as *Faecalibacterium prausnitzii* type G and *Bacteroides uniformis* with a prevalence of ≥87.5%. The remaining species are shown in Figure 5.

In patients with CD at diagnosis, only four taxa were present in more than 50% of cases: *Bacteroides vulgatus* (90%), *Blautia wexlerae* (70%), *Bacteroides uniformis* (60%), and *Bacteroides ovatus* (60%).

Core microbiome analysis at follow-up identified five taxa present in more than 50% of patients: *Bacteroides vulgatus* (100%), *Faecalibacterium prausnitzii* type G (80%), *Blautia wexlerae* (80%), *Bacteroides ovatus* (80%), and *Parabacteroides distasonis* (60%).

**Figure 6:**
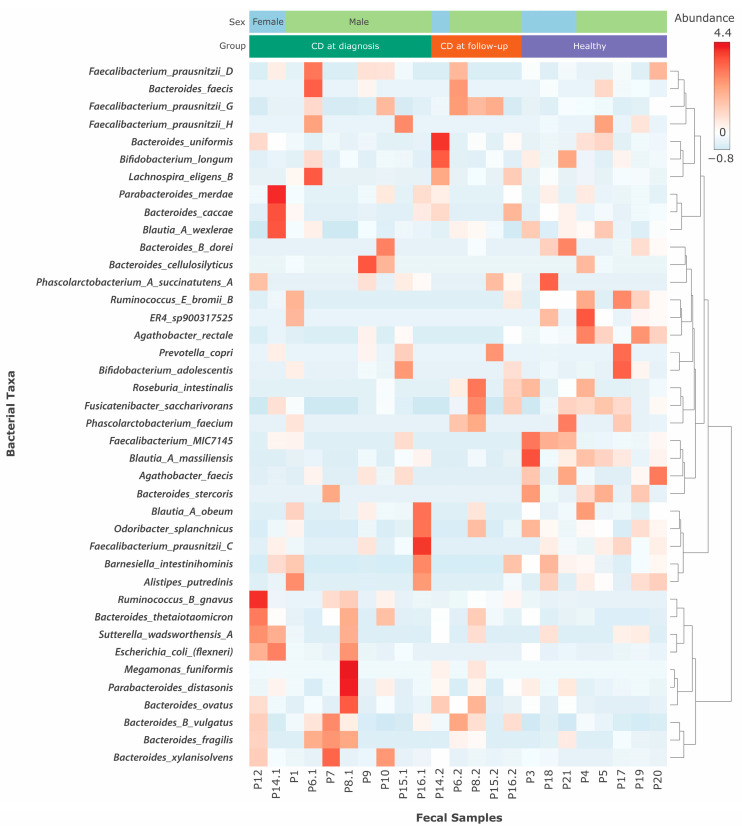
Heatmap showing sample clustering according to group (CD at diagnosis, CD at follow-up, healthy). Color intensity indicates the relative abundance of taxa. Dendrograms show hierarchical clustering based on taxon abundance profiles.

The heatmap revealed a distinct clustering of samples according to group, as shown in Figure 6. Most healthy individuals formed a separate cluster, characterized by higher abundance of commensal species such as *Ruminococcus bromii*, *Agathobacter rectale*, *Agathobacter faecis*, *Fusicatenibacter saccharivorans*, *Faecalibacterium* MIC7145, *Blautia massiliensis*, and *Bacteroides stercoris*.

In contrast, samples from patients with CD displayed a more heterogeneous pattern, with higher abundance of taxa such as *Ruminococcus gnavus* and *Escherichia coli*, consistent with intestinal dysbiosis, as well as species such as *Bacteroides vulgatus*, *Bacteroides fragilis*, and *Sutterella wadsworthensis*, which may exhibit both commensal and opportunistic behavior.

Differential analysis using LEfSe identified taxa contributing to between-group differences. *Blautia massiliensis*, *Agathobacter rectale*, and *Faecalibacterium* MIC7145 were more closely associated with the healthy group (FDR < 0.065), whereas *Ruminococcus gnavus* and *Escherichia coli* were more abundant in patients with CD at diagnosis.

**Figure 7:**
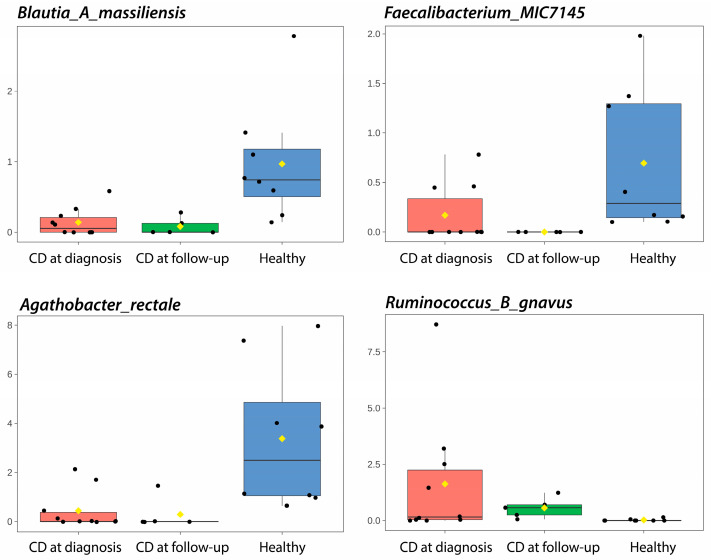
Distribution of filtered relative abundance values for selected taxa across the study groups. Box plots show median and interquartile range; individual points represent samples; yellow diamonds indicate mean values.

Additional taxa showed nominally significant *p*-values but borderline FDR values, suggesting possible taxon-level differences. Complete data for the main taxa are presented in Table 3. To illustrate the differences identified by LEfSe analysis, box plots were generated based on filtered data, highlighting the distribution of relative abundance for relevant taxa across the analyzed groups, as shown in Figure 7.

The Random Forest model, based on taxon-level features, showed an OOB error of 43.5%. The model correctly classified 75% of healthy controls and 70% of patients with CD at diagnosis.

Samples from patients with CD at follow-up could not be reliably distinguished from those at diagnosis, suggesting a microbial profile similar to the active disease state. At the same time, these samples were not misclassified as healthy, indicating no clear shift toward a microbiota composition comparable to that of healthy individuals.

**Figure 8:**
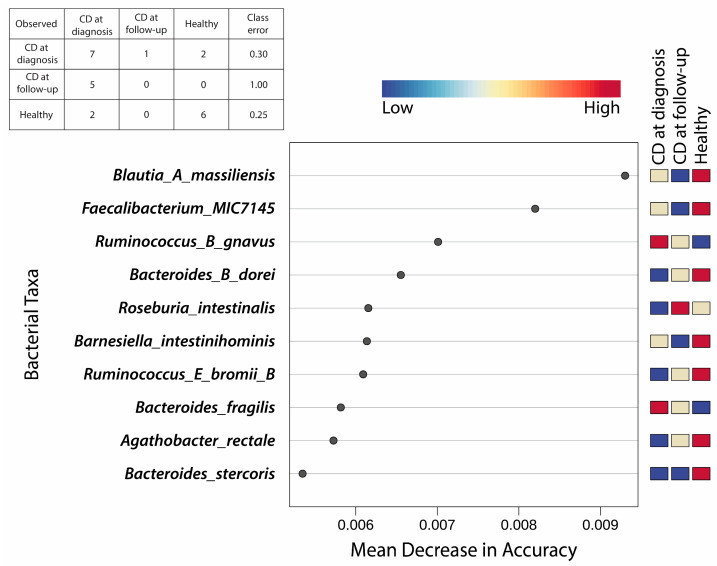
Variable importance plot of taxa identified by the Random Forest model, based on the Mean Decrease Accuracy score. The table represents the out-of-bag (OOB) classification matrix, including classification errors for each group.

The variable importance plot showed that *Blautia massiliensis* was the highest-ranking taxon in this exploratory model, followed by *Faecalibacterium* MIC7145 and *Ruminococcus gnavus*, as illustrated in Figure 8.

### 3.3. Intra-Individual Microbial Analysis

Comparison of the Shannon index in patients with CD at diagnosis and at follow-up showed no statistically significant difference between the two time points (V = 12, *p* = 0.3125), suggesting similar alpha diversity over the follow-up period.

**Figure 9:**
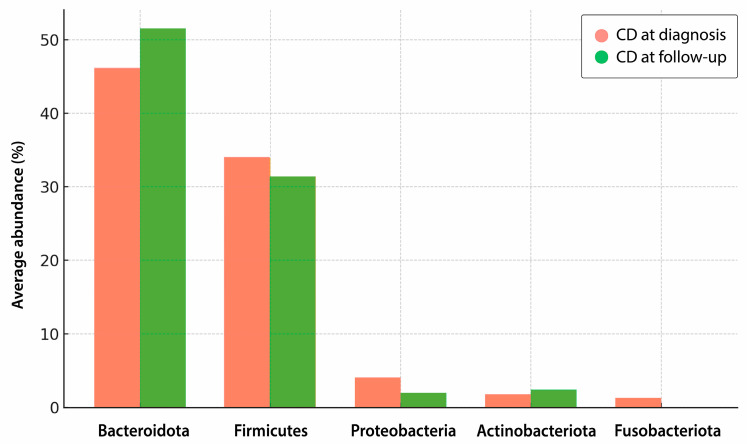
Mean relative abundance at the phylum level in patients with CD at diagnosis and at follow-up.

Mean relative abundances of the main bacterial phyla were compared between diagnosis and follow-up. The comparative plot of mean values (Figure 9) indicates a relatively stable overall structure, with slight variations in the proportions of Bacteroidota (46% at t(0) vs. 51.5% at t(1)) and Firmicutes (33.9% at t(0) vs. 31.4% at t(1)). Other phyla, such as Proteobacteria, Actinobacteriota, and Fusobacteriota, showed low abundance and non-significant variation. Paired Wilcoxon test analysis did not reveal statistically significant differences between the two time points for any of the included phyla (*p* > 0.28). These findings suggest a generally similar bacterial composition over the follow-up period.

To assess changes at the level of dominant bacterial taxa, paired relative abundances were compared. No statistically significant differences were identified, with *p*-values > 0.05 in all cases. However, *Bacteroides vulgatus* showed a trend toward increased abundance (mean difference: 0.0968; *p* = 0.0625), while *Escherichia coli* showed a decrease (mean difference: 1.62; *p* = 0.100). Notably, *E. coli* was completely absent in all samples at follow-up.

**Figure 10:**
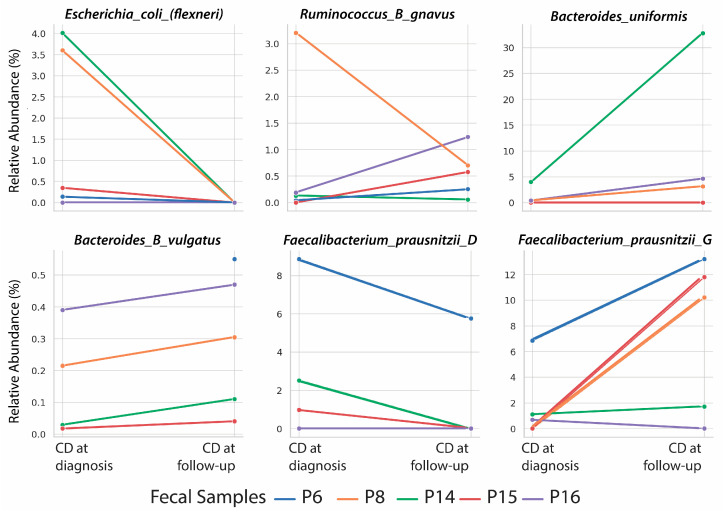
Evolution of relative abundance (%) of selected taxa in patients with CD at diagnosis and follow-up. Each line represents an individual patient.

For the remaining taxa analyzed, including *Bacteroides uniformis*, *Faecalibacterium prausnitzii* (types D and G), and *Ruminococcus gnavus*, only minor mean differences were observed, with non-significant *p*-values (*p* > 0.18). The plots generated for these taxa (Figure 10) illustrate intra-individual variability: some samples showed slight fluctuations over time, without a consistent pattern of increase or decrease. These findings suggest limited taxon-level changes in the microbial community structure at follow-up.

## 4. Discussion

Bacterial diversity represents a central aspect investigated in CD, providing direct insight into the state of dysbiosis. In the present study, children with newly diagnosed CD, presenting with non-complicated phenotypes despite variable clinical severity at diagnosis, exhibited significantly reduced alpha diversity compared to healthy children. In addition, beta diversity analysis revealed structural differences between patients and healthy controls, reflecting a disrupted intestinal microbial community. These findings are consistent with the existing literature, which describes reduced diversity and altered microbiota structure in CD [7,24,25].

Furthermore, alpha diversity assessed by the Shannon index did not show significant correlations with inflammatory markers (CRP, ESR, FC) or BWT, but demonstrated an inverse association with the wPCDAI score. This finding may suggest that microbial diversity is more closely related to overall clinical disease activity than to isolated inflammatory biomarkers or imaging parameters. These results are partially consistent with previous studies, which have not reported consistent correlations between alpha diversity and FC, but have described negative associations between the Shannon index and clinical disease activity, particularly in ulcerative colitis [26].

At the phylum level, the present analysis showed a significant decrease in the abundance of Firmicutes and a significant increase in Bacteroidota in patients with CD compared to healthy controls. A slight increase in Proteobacteria abundance was also observed, while Actinobacteriota did not show significant variation. This pattern partially differs from previous reports describing reduced Firmicutes and increased Proteobacteria [27,28]. A recent systematic review in pediatric CD reported decreased Actinobacteria and Bacteroidota and a marked increase in Proteobacteria, consistent with adult data [24]. These discrepancies may reflect the influence of factors such as sample size and cohort-specific characteristics.

*Ruminococcus gnavus* is a Gram-positive anaerobic bacterium belonging to the phylum Firmicutes, taxonomically classified within the genus *Mediterraneibacter*. It is commonly found in the human gut, with high abundance in neonates and young children (up to 83%), lower levels in adults, and a slight increase in the elderly, with higher prevalence in Westernized populations; breastfeeding has been shown to significantly reduce colonization in infants [29]. The significance of *R. gnavus* in IBD has long been underestimated due to initial misclassification and limitations of 16S rRNA sequencing, which does not allow for accurate species-level discrimination. Consequently, decreases in other *Ruminococcus* species may have masked increases in *R. gnavus* when analyses were performed at the genus level.

In CD, *R. gnavus* has frequently been reported at increased levels [30,31,32] and has been associated with a more extensive disease phenotype [32], although some studies have paradoxically linked its presence to a more favorable prognosis [30]. Recent studies have identified distinct genomic clades of *R. gnavus* carrying CD-associated genes related to oxidative stress resistance, suggesting an adaptive advantage in inflamed gut conditions [29]. Certain strains also exhibit mucolytic activity by utilizing glycans from the intestinal mucus layer, potentially disrupting mucus barrier integrity [33]. This may facilitate closer interaction between luminal bacteria and the epithelium and promote colonization by pathobionts such as adherent-invasive *Escherichia coli* (AIEC), thereby contributing to persistent mucosal inflammation. In addition, *R. gnavus* can produce pro-inflammatory polysaccharides that may further amplify inflammatory responses [34]. In this context, increased abundance of *R. gnavus* has been frequently reported in CD, with transient peaks observed during periods of active disease [35,36] suggesting a possible association between its expansion and disease activity.

Consistent with these findings, *R. gnavus* was significantly more abundant in patients with CD at diagnosis in the present study, as demonstrated by both differential abundance analysis and LEfSe. It was also identified among the taxa contributing most to group separation in the Random Forest model. However, this observation should be interpreted cautiously given the limited classification performance of the model (OOB error of 43.5%). In contrast, Gevers et al. reported decreased *R. gnavus* abundance in treatment-naïve pediatric CD patients using metagenomic analysis of fecal samples [6].

*Escherichia coli* is a commensal bacterium of the human gut microbiota; however, certain strains (AIEC) acting as pathobionts have been implicated in CD. AIEC strains are characterized by their ability to adhere to and invade intestinal epithelial cells, survive and replicate within macrophages, and induce chronic inflammation [37,38]. These strains preferentially colonize the terminal ileum and are implicated in the early stages of ileal CD [39,40]. Previous studies have shown a positive association between pathogenic *E. coli* and ulcer formation through specific functional bacterial mechanisms [6]. Patients with CD frequently exhibit increased abundance of Proteobacteria and *E. coli* compared to healthy individuals [6,30,31,36]. In the present study, *E. coli* was more abundant in patients with CD at diagnosis compared to healthy controls, suggesting a potential role in active inflammatory processes.

Species belonging to the genus *Faecalibacterium* are metabolically versatile microorganisms and are among the most abundant and important commensals in the healthy human gut, known for their anti-inflammatory properties [41]. *Faecalibacterium prausnitzii* contributes to the maintenance of intestinal barrier integrity and immune regulation through the production of short-chain fatty acids, particularly butyrate [42]. In CD, numerous studies have reported a significant decrease in *F. prausnitzii* abundance in both intestinal biopsies and fecal samples [6,24,43,44,45]. However, this pattern is not universal; Hansen et al. reported increased *F. prausnitzii* abundance in children with colonic CD at onset based on mucosal biopsy analysis [46]. Prior antibiotic exposure has been associated with reduced levels of this taxon [32]. McLellan et al. observed that *F. prausnitzii* was the only *Faecalibacterium* species showing an increase from disease flare to remission, although without statistical significance [41].

In our study, total *F. prausnitzii* abundance did not differ significantly between groups. However, differences were observed at subtype level. *F. prausnitzii* type G was identified within the core microbiome of both healthy children and patients with CD at follow-up, whereas paired analysis did not show significant changes in types D and G between diagnosis and follow-up. In contrast, LEfSe analysis suggested that type C was detected in healthy children and patients with CD at diagnosis, but was not identified at follow-up. In addition, *Faecalibacterium* MIC7145 was more abundant in healthy controls. These findings suggest subtype-specific restructuring within the *Faecalibacterium* community, whereby increases or decreases in individual subtypes may be masked when only total *F. prausnitzii* abundance is considered. Therefore, similar overall levels of *F. prausnitzii* do not necessarily indicate preservation of the same microbial composition or functional potential. However, given the small sample size and borderline FDR values, these subtype-level findings should be interpreted as exploratory and require validation in larger cohorts. The subdivision of *Faecalibacterium* spp. may partly explain differences between studies using distinct taxonomic classification approaches. In this context, future studies should investigate not only total *F. prausnitzii* abundance, but also subtype-level dynamics within the *Faecalibacterium* community, in order to better define their potential roles in CD-associated dysbiosis.

The microbial core of healthy children included key taxa such as *Agathobacter rectale*, *Blautia wexlerae*, *Ruminococcus bromii*, and *Faecalibacterium* spp., which are known for their beneficial roles in maintaining intestinal homeostasis through butyrate production, maintenance of intestinal barrier integrity, and modulation of inflammatory responses. LEfSe analysis also showed that *Blautia massiliensis*, *Agathobacter rectale*, and *Faecalibacterium* MIC7145 were more closely associated with the healthy group, suggesting their role as potential indicators of eubiosis [47,48]. In addition, members of the phylum Bacteroidota, including *Bacteroides uniformis*, *B. vulgatus*, *B. stercoris*, and *B. caccae*, contributed to this core community. They are involved in key functions such as complex polysaccharide degradation, short-chain fatty acid production, and the maintenance of intestinal immune and metabolic homeostasis [49].

In the present study, all re-evaluated patients with CD received a nutritional intervention (namely, CDED), preceded in 4/5 cases by short-term EEN. One patient received CDED combined with partial enteral nutrition as sole induction therapy, while 2/5 patients received corticosteroids and 2/5 received 5-ASA. At follow-up, all patients had achieved CR, while the majority (80%) attained BR and MH; IR and TH were observed in a substantial proportion of cases (60% and 40%, respectively). Despite these favorable outcomes, patients continued to exhibit a dysbiotic gut profile, with persistently reduced alpha diversity and significant compositional differences compared with healthy controls. Core commensal species such as *B. longum*, *B. caccae*, and *B. fragilis* remained less abundant, without statistical significance, suggesting partial but incomplete restoration of eubiosis. Notably, *F. prausnitzii* type G was present in the microbial core of 80% of patients in remission. *E. coli* showed a marked decrease, disappearing entirely in all samples at follow-up. Furthermore, *Roseburia intestinalis*, a butyrate-producing taxon, exhibited increased abundance at follow-up compared to diagnosis; however, this finding did not remain statistically significant after FDR correction.

These findings contribute to understanding intestinal microbiota dynamics under treatment and support the notion that microbial profiles may remain altered even in CR, as also reported in the recent literature [50].

CDED is a dietary intervention combined with partial enteral nutrition, typically implemented over approximately 12 weeks. It was designed to exclude or limit dietary components that are potentially pro-inflammatory, including ultra-processed foods, emulsifiers, maltodextrin, animal fats, and processed meats, while encouraging whole, minimally processed foods [16,51]. Published data on CDED showed similar shifts in microbial composition, particularly a reduction in Proteobacteria, including *Escherichia coli*, alongside increases in selected Firmicutes taxa such as *Roseburia* and *Ruminococcus*. These changes appear to accompany clinical improvement; however, the microbial profile does not fully resemble that of healthy individuals even after clinical remission is achieved [50]. A possible explanation is that reduction in pro-inflammatory dietary exposures may lead to an early decline in inflammation-related microorganisms, whereas broader restructuring of the intestinal microbial ecosystem likely requires longer-term or sustained intervention. In addition, the increase in Firmicutes may partly reflect ecological redistribution within the microbial community following the reduction in Proteobacteria, rather than complete microbial recovery.

The primary limitation of the present study relates to the small sample size, particularly in the longitudinal component, which reduces the robustness and generalizability of the findings and precluded subgroup analyses according to treatment type. This is particularly relevant given the heterogeneity of induction therapies used in this cohort, which may influence the intestinal microbiota through distinct mechanisms. Dietary therapies such as CDED and EEN are thought to directly modulate microbial composition by altering nutrient availability and reducing exposure to pro-inflammatory dietary components [8,50,51], whereas corticosteroids primarily reduce intestinal inflammation and may indirectly affect microbial communities through changes in the inflammatory environment. In addition, 5-ASA has been associated with modulation of bacterial biofilm formation and alterations in selected bacterial taxa [52], while antibiotics such as azithromycin may induce direct shifts in microbial composition. Consequently, treatment-related microbial changes may have contributed to the observed inter-individual variability, limiting attribution of microbiota changes exclusively to disease activity, and should therefore be taken into account when interpreting associations between microbial patterns and clinical outcomes.

The limited cohort size should be interpreted in the context of the low incidence of pediatric IBD in the North-West region of Romania [53]. The partial overlap with the COVID-19 pandemic may have influenced patient access to healthcare and follow-up regularity.

Another important limitation of this study is the single-center design, which may reflect cohort-specific characteristics related to local clinical practice. Nevertheless, adherence to ESPGHAN guidelines supports comparison with other international pediatric cohorts.

Microbiota analysis was based exclusively on fecal samples, without mucosal sampling, which may limit characterization of microbiota directly associated with intestinal lesions. In addition, the analysis focused primarily on taxonomic composition and did not include functional omics approaches, such as metabolomic or transcriptomic profiling, thereby limiting insight into the functional consequences of the observed microbial alterations in pediatric CD. The limited number of sampling time points further restricted detailed assessment of microbiota dynamics, partly due to the high cost of metagenomic sequencing. Therefore, all these limitations should be considered when interpreting the biological and clinical significance of the observed microbial alterations.

Despite these limitations, the study has several important strengths. Firstly, its prospective design with standardized longitudinal follow-up enabled assessment of gut microbiota patterns after induction of remission. Secondly, treatment outcomes were evaluated using multiple parameters, including CR, BR, MH, and TH. Thirdly, the use of metagenomic sequencing represents a major methodological strength, providing higher taxonomic resolution and more comprehensive microbial profiling than conventional 16S rRNA-based approaches, including species-level identification of relevant taxa. Finally, the study provides novel data in an understudied pediatric population from Eastern Europe, where microbiome data in CD remain limited.

Future research should prioritize larger, multicenter pediatric CD cohorts with standardized treatment protocols, repeated longitudinal microbiota sampling, and integration of functional omics approaches. Such studies would provide a more robust framework for characterizing microbiota dynamics during remission induction and follow-up, distinguishing disease-associated from treatment-related microbial changes, and determining the clinical relevance of persistent alpha diversity reduction and taxon-specific alterations, including those involving *R. gnavus* and *Faecalibacterium* subtypes.

## 5. Conclusions

Through an analysis of the gut microbiota in pediatric patients with CD from Romania, the present study confirmed the presence of marked dysbiosis at diagnosis. At follow-up, although all patients were in CR and the majority had BR and MH, the microbial composition did not return to a profile comparable to that of healthy children, with persistently reduced alpha diversity and taxonomic imbalances. Some butyrate-producing taxa showed higher abundance at follow-up, although these findings should be interpreted cautiously given the small longitudinal subgroup. The inverse correlation between the Shannon index and the wPCDAI score suggests a possible association between reduced alpha diversity and disease severity.

## Figures and Tables

**Table 1:** Distribution of patients with CD according to disease characteristics based on the Paris classification [17].

Items	Subtype	Number of Patients (%)
Age at diagnosis (A)	A1a (<10 years old)	1 (10%)
	A1b (10–16 years old)	8 (80%)
	A2 (>17 years old)	1 (10%)
Location (L)	L1 (ileal)	3 (30%)
	L2 (colonic)	2 (20%)
	L3 (ileocolonic)	5 (50%)
	L4 (upper digestive tract)	2 (20%)
	L4a (proximal to Treitz ligament)	2 (20%)
	L4b (distal to Treitz ligament)	1 (10%)
Behavior (B)	B1 (inflammatory)	10 (100%)
	B2 (stricturing)	0 (0%)
	B3 (penetrating)	0 (0%)
	B2B3 (stricturing + penetrating)	0 (0%)
	Perianal disease modifier (p)	0 (0%)
Growth (G)	G0 (absence of growth delay)	8 (80%)
	G1 (growth delay)	2 (20%)

**Table 2:** Relative abundance of the main bacterial phyla in patients with Crohn’s disease (CD) at diagnosis (t(0)), at follow-up (t(1)), and in healthy children.

Phylum	CD at t(0) (%)	CD at t(1) (%)	Healthy (%)	*p* Value
Firmicutes	32.7	31.4	45.9	0.030
Bacteroidota	49.1	51.5	36.4	0.016
Actinobacteriota	1.4	2.3	2.92	0.683
Proteobacteria	3.23	1.9	1.8	0.542

**Table 3:** Results of LEfSe analysis (Linear Discriminant Analysis Effect Size): differential taxa between groups, with *p*-values, false discovery rate (FDR), and LDA (Linear Discriminant Analysis) scores. Columns represent the mean relative abundance of each taxon across the analyzed groups.

	*p* Value	FDR	CD t(0)	CD t(1)	Healthy	LDA
Firmicutes_A*_Blautia_A_* *massiliensis*	0.002	0.065	0.139	0.081	0.968	0.159
Firmicutes_A*_Faecalibacterium_MIC7145*	0.004	0.065	0.169	0.000	0.694	0.129
Firmicutes_A*_Agathobacter_* *rectale*	0.004	0.065	0.444	0.294	3.381	0.405
Firmicutes_A*_Ruminococcus_* *B_gnavus*	0.012	0.123	1.627	0.565	0.025	0.256
Bacteroidota*_Bacteroides_* *stercoris*	0.025	0.151	0.492	0.000	2.049	0.306
Firmicutes_A*_ER4_* *sp900317525*	0.025	0.151	0.128	0.000	0.562	0.108
Firmicutes_A*_Faecalibacterium_prausnitzii_C*	0.028	0.151	0.703	0.000	0.640	0.131
Proteobacteria*_Escherichia_coli_(flexneri)*	0.031	0.151	1.124	0.000	0.104	0.194
Firmicutes_A*_Ruminococcus_E_bromii_B*	0.037	0.151	0.400	0.332	1.759	0.234
Firmicutes_A*_Agathobacter_* *faecis*	0.038	0.151	0.583	0.000	1.872	0.287
Firmicutes_A*_Roseburia_* *intestinalis*	0.0417	0.1516	0.0215	0.6198	0.3425	0.114

## Data Availability

The original contributions presented in this study are included in the article; further inquiries can be directed to the corresponding author, within the legal regulations.
